# Intravascular Photothermal Strain Imaging for Lipid Detection

**DOI:** 10.3390/s18113609

**Published:** 2018-10-24

**Authors:** Changhoon Choi, Joongho Ahn, Chulhong Kim

**Affiliations:** Departments of Creative IT Engineering, Pohang University of Science and Technology (POSTECH), Pohang 37673, Korea; chang8909@postech.ac.kr (C.C.); joongho.ahn@postech.ac.kr (J.A.)

**Keywords:** cardiovascular disease, unstable plaque, intravascular imaging, thermal strain imaging, intravascular ultrasound (IVUS), photothermal strain imaging, lipid detection

## Abstract

Cardiovascular disease (CVD) is one of the major threats to humanity, accounting for one-third of the world’s deaths. For patients with high-risk CVD, plaque rupture can lead to critical condition. It is therefore important to determine the stability of the plaque and classify the patient’s risk level. Lipid content is an important determinant of plaque stability. However, conventional intravascular imaging methods have limitations in finding lipids. Therefore, new intravascular imaging techniques for plaque risk assessment are urgently needed. In this study, a novel photothermal strain imaging (pTSI) was applied to an intravascular imaging system for detecting lipids in plaques. As a combination of thermal strain imaging and laser-induced heating, pTSI differentiates lipids from other tissues based on changes in ultrasound (US) velocity with temperature change. We designed an optical pathway to an intravascular ultrasound catheter to deliver 1210-nm laser and US simultaneously. To establish the feasibility of the intravascular pTSI system, we experimented with a tissue-mimicking phantom made of fat and gelatin. Due to the difference in the strain during laser heating, we can clearly distinguish fat and gelatin in the phantom. The result demonstrates that pTSI could be used with conventional intravascular imaging methods to detect the plaque lipid.

## 1. Introduction

Cardiovascular disease (CVD) is one of the major threats to humanity. According to a report by the American Heart Association, CVD accounts for one-third of the world’s deaths, and the leading cause of death in CVD patients is coronary heart disease (CHD) [1]. Rupture of high-risk plaques is associated with intravascular thrombus formation, which can lead to acute CHD such as myocardial infarction or stroke [2,3]. Lipid content is one of the important determinants of plaque stability. Many studies have shown that the size of the lipid pool plays an important role in determining the stability of the plaque [4,5,6]. In addition, lipid concentration of the plaque is related to the thickness of the fibrous cap and the number of macrophages, which are other important indicators of plaque stability [7]. Moreover, lipids are involved in the formation of cholesterol crystals that can cause plaque rupture by tearing the fibrous cap [8,9]. Therefore, measuring the plaque lipid is important in assessing the risk of CVD patients.

Recently, several intravascular imaging techniques in commercial or experimental stages have been used for plaque imaging. Intravascular ultrasound (IVUS) visualizes the morphological structure of blood vessels, but it is difficult to distinguish lipids to assess plaque stability [10]. Virtual histology intravascular ultrasound (VH-IVUS) provides information on tissue types based on IVUS signal analysis, but clinical efficacy for plaque analysis has not been fully validated [11,12]. Intravascular optical coherent tomography (IV-OCT) provides a high-resolution superficial image, which is sufficient to distinguish the thickness of plaque fibrous cap [13,14]. However, IV-OCT suffers from low imaging depth and it is difficult to distinguish the plaque components. Intravascular photoacoustic (IVPA) imaging [15], which is currently being actively studied, can provide physiological information based on optical properties of tissue and is used in combination with IVUS or IV-OCT to provide both structure and information of tissues simultaneously [16,17]. However, it is difficult to obtain a 1210-nm or 1720-nm pulsed laser source necessary for lipid imaging. Many groups are working to develop new laser sources for lipid imaging, but they are still expensive, voluminous, and not readily available [18,19,20]. Near-infrared spectroscopy (NIRS) is an imaging method based on chemical sensing that can detect the presence of lipids very accurately [21,22]. However, NIRS cannot quantify the depth of the lipid because depth resolution is not available. Near-infrared fluorescence (NIRF) can detect the presence of lipids with high accuracy and is used with IVUS or IV-OCT to provide structural information [23,24]. However, the clinical use of NIRF is limited because of its low depth resolution and the use of chemicals. Near-infrared auto-fluorescence (NIRAF) is an auto-fluorescence-based technique that can accurately locate lipids without the use of chemicals, but low depth sensitivity is still a problem [25,26]. Therefore, to overcome the limitations of current technology, new techniques are needed to image and quantify the plaque lipid.

In our previous study, we introduced a photothermal strain imaging (pTSI) as a new imaging technique for lipid detection [27]. pTSI, a fusion of thermal strain imaging (TSI) and laser-induced heating, uses changes in ultrasound (US) velocity associated with medium temperature changes. This velocity change causes speckle shifts in the US image, which can be used to estimate the strain between two US images [28]. Because the parameters of US velocity vary from tissues to tissues, analyzing the generated strain can infer the type of tissues [29]. Conventional TSI methods use heat sources such as microwaves [30] or US [31,32], while pTSI uses a relatively inexpensive, readily available continuous-wave (CW) laser as a heat source. In addition, pTSI allows the laser to pass through the optical fiber to directly heat the target in the body. This direct heating of a localized area can minimize unnecessary heating. Therefore, pTSI has higher heating efficiency and less patient burden than the conventional method.

In this study, pTSI was applied to an intravascular imaging system for detection of the plaque lipid. To transmit light and US simultaneously into the blood vessel, we developed a catheter with an optical pathway over the conventional IVUS and used a 1210-nm CW laser as the heat source for pTSI. Because lipids and water strongly absorb light with a wavelength of 1210-nm [33], lipids and surrounding water-bearing tissues can heat up by the laser relatively quickly than other tissues. To demonstrate the ability of the intravascular pTSI system to detect lipids, we performed pTSI experiments with tissue-imitated phantom made of gelatin and porcine subcutaneous fat. The next few sections describe the system and experimental settings and provide results and conclusions.

## 2. Materials and Methods

### 2.1. Photothermal Strain Imaging for Tissue Characterization

The theoretical basis of pTSI is the velocity change of the US wave due to a temperature change of medium. Changes in US velocity will shift the echoes of the scatterers in the US image. At small temperature changes near room temperature thermal expansion can be ignored [34], so the scatterers themselves do not move and only the speckle shift occurs in the US image. Thermal strain, also called temporal strain, can be expressed using Equation (1):

(1) $$Thermal\;strain=\;\frac{\delta }{\delta t}\left[\delta t\left(x_{0}\right)\right]≅\left[\beta \left(x_{0}\right)-\lambda \left(x_{0}\right)\right]\delta \theta \left(x_{0}\right)$$

where *x*_0_ is the depth of tissue, *δt*(*x*_0_) is the round-trip time delay of an echo from the scatterer at *x*_0_, *β* is the linear coefficient of thermal expansion, *λ* is the linear coefficient of US velocity change against temperature variation, and *δθ*(*x*_0_) is the temperature change in the tissue [35]. Equation (1) can be approximated as a function of *λ* and *δθ* because *β* is very small compared to *λ* for small temperature changes in soft tissue. Thus, when the temperature changes, the strain changes based on the difference in *λ* between tissues, so we can infer the tissue type based on the strain. Lipids we target have a negative *λ* that produces a positive strain when the temperature rises, while water-bearing tissues have a positive *λ* that produces a negative strain when the temperature rises [36]. Therefore, the positive strain generating in lipids can be distinguished from the negative strain in water-bearing tissues.

Instead of conventional heat sources such as microwaves or US used in TSI, pTSI uses CW lasers as a heat source. Since each tissue has different optical properties, the laser absorption of tissue depends on the wavelength of the laser used. Therefore, depending on the situation, pTSI can choose the laser of a specific wavelength that can be strongly absorbed by the target tissues to minimize the temperature change of the nontarget tissues. In addition, the laser is an efficient heating method in that it can enter the body through the optical fiber and directly heat the target. Finally, unlike conventional methods of heating a large area, the laser can heat the target site locally, reducing the burden on patients. Based on these advantages, we expect pTSI to be more flexible in clinical situations than TSI.

### 2.2. Intravascular pTSI Catheter and Experimental Setup

**Figure 1:**
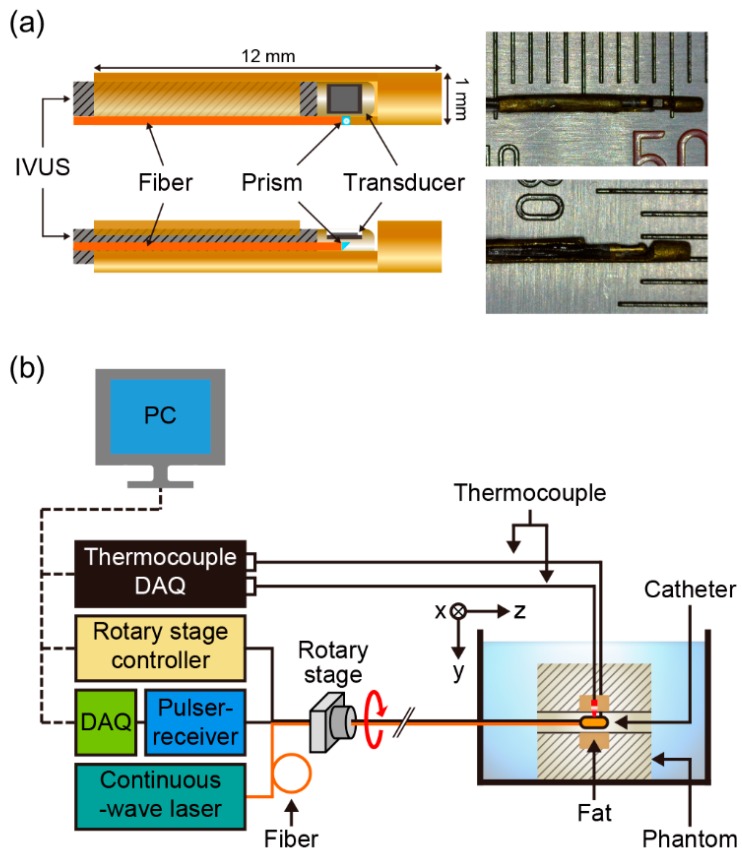
(**a**) Schematics and photographs of an intravascular photothermal imaging (pTSI) catheter. (**b**) A schematic of the intravascular pTSI experimental setup. DAQ: data acquisition system.

Figure 1a shows schematics and photographs of the intravascular pTSI catheter. A single-element transducer with an element size of approximately 0.4 mm × 0.5 mm, a center frequency of 41.7 MHz, and a −6 dB bandwidth of 35.5% on a conventional IVUS catheter (Opticross^TM^, Boston Scientific, Marlborough, MA, USA) was used to acquire US images. The resolution of the US image was obtained from a 6-μm carbon fiber at a distance of 3 mm and was measured 57-μm in the axial direction and 269-μm in the lateral direction. The IVUS tip with transducer was fixed to a brass catheter head with a length of 12 mm and a diameter of 1 mm. An optical fiber combined with a right-angle prism was fixed next to the transducer so that the laser illuminated where US images were obtained.

Figure 1b shows a schematic of the intravascular pTSI experimental setup. The pTSI catheter was inserted into the hole in the phantom placed in a water tank. A rotary stage (ST-HB-60-5-ST-PR-PT, Science Town, Incheon, Republic of Korea) under the control of a controller (HRT60-05-I, Science Town, Incheon, Republic of Korea) rotated the pTSI catheter. A US pulser-receiver (5073PR, Olympus, Tokyo, Japan) generated US signal through the IVUS transducer and amplified the received US signal reflected from the target. A 14-bit digitizer (Razor 1422, Gage, Lockport, IL, USA) in the data acquisition system (DAQ) received the US radio frequency (RF) data from the pulser-receiver at a sample rate of 200 MS/s and stored it on a PC. Fluoroptic thermocouples (STB, Luxtron, Santa Clara, CA, USA) under the control of a thermocouple DAQ (m600, Luxtron, Santa Clara, CA, USA) were connected to gelatin and fat to monitor and recorded temperature data. A compact size (240 mm × 150 mm × 120 mm) 1210-nm CW laser (ALC-1210-04000-CB100-LDTC, Akela, Jamesburg, NJ, USA) was used as the heat source. The laser was directed through the multimode fiber to the catheter head and illuminated the target. Since it was found that the tendency of changes in US velocity during the rise of the temperature of fat and water-bearing tissues was not different at the room and body temperatures [29], all experiments were performed at the room temperature where gelatin was not dissolved. In all experiments, we strictly adhered to the laser safety limits of human skin for long time exposure (<1 W/cm^2^) as specified in the ANSI safety standard (ANSI Z136.1).

### 2.3. Data Acquisition and Imaging Processing

**Figure 2:**
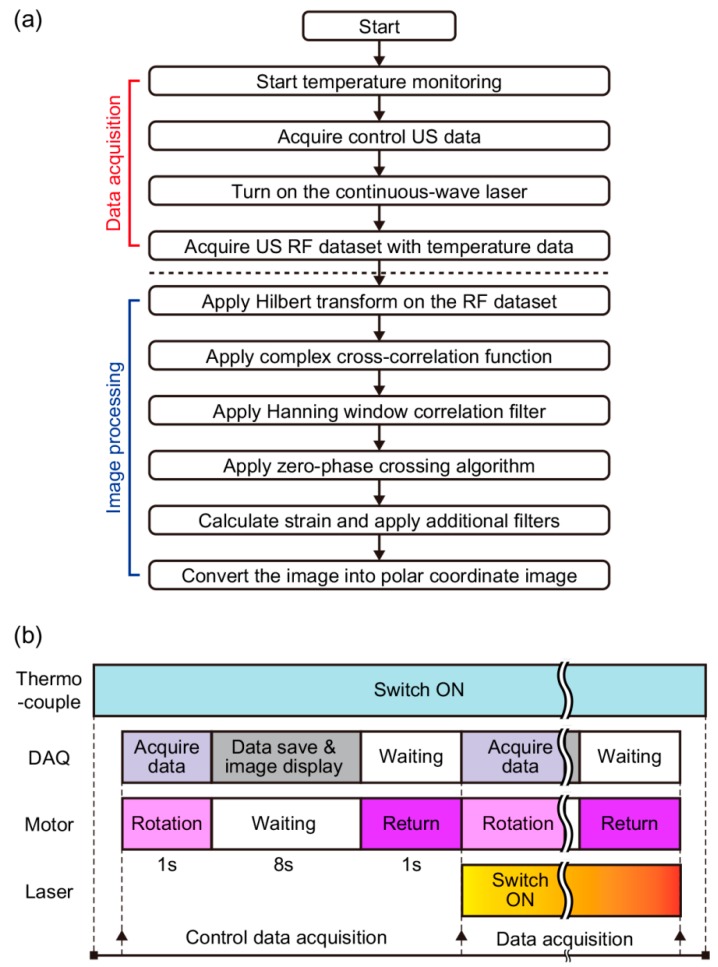
(**a**) A block diagram of data acquisition and image processing part for intravascular photothermal strain imaging. (**b**) Timing diagrams of thermocouple, data acquisition system (DAQ), rotary stage motor, and laser operation. US, ultrasound; RF, radio-frequency.

Figure 2a shows the complete sequence of pTSI data acquisition and image processing. Figure 2b shows the timing diagrams of the thermocouple, the DAQ, the rotary stage motor, and the laser in data acquisition. From the beginning to the end of the experiment, the thermocouples constantly monitored the temperature of the target. When collecting data, the catheter only rotated once at a rate of one rotation per second (RPS) and counter-rotated to its original position to minimize position errors due to inconsistencies in the starting positions. The laser began to heat the target for one minute after the US data of the control condition (i.e., the data obtained with the laser turned off) was obtained. Then, we repeatedly collected the US data every 10 s while the laser was on. During the experiment, DAQ stored US RF data in binary format on the PC and displayed the US image in polar coordinates via LabVIEW (National Instruments, Austin, TX, USA). At the end of the experiment, we were able to collect eight US images.

After the experiment, we applied a speckle tracking algorithm to the US data to obtain strain images [37]. First, the Hilbert transform enveloped the US RF data. At this stage, a 2D cross-correlation-based position-correction algorithm was applied to match the lateral starting positions of the US B-mode images. Then, the normalized complex cross-correlation function estimated the displacements by calculating the maximum correlation coefficient at the same location of different data. The autocorrelation of US RF signals determined the proper kernel size (150 μm) used for the cross-correlation. During the cross-correlation process, correlation filters used the Hanning window that was 1.5 times the size of the cross-correlation kernel to average the correlation result to reduce peak hopping errors. Next, the zero-phase crossing algorithm was used to more precisely sample the displacements. Based on the obtained displacements, the initial strain was calculated. The median filter (which was 1.5 times the size of the cross-correlation kernel) further filtered the strain result to reduce residual errors [32], while the Gaussian function (with a standard deviation of 2) smoothed the strain edges. Finally, we superimposed the strain on the US image in polar coordinates and were able to see where the strain occurred. All postprocessing algorithms were processed using MATLAB (MathWorks, Natick, MA, USA).

### 2.4. Tissue-Mimicking Phantom for pTSI

We prepared the tissue-mimicking phantom as an image sample for intravascular pTSI experiments [38,39]. The phantom was made of two parts: one was water-bearing tissue made from a solution of 10% gelatin and 20% cornstarch (by weight), and the other was porcine subcutaneous fat. First, we added the cornstarch to water at room temperature to create a speckle pattern in the phantom US image. Next, gelatin powder (G2500, Sigma-Aldrich, St. Louis, MO, USA) was added to the solution little by little. It is recommended not to add a lot of cornstarch or gelatin powder at one time to avoid an aggregation of powder. A magnetic stirrer with a heat pad stirred and heated the mixed solution until the solution sufficiently dissolved the gelatin powder. Then, we poured the completely mixed solution into a custom-designed frame. At this stage, two pieces of porcine fat with 4 mm × 4 mm × 30 mm along x, y, and z axes, respectively, were attached to the top and bottom of the sample hole that had a 3-mm diameter. The solution was solidified in a refrigerator for one day. Finally, we took out the phantom from the frame and made the phantom into the block of size 30 mm × 55 mm × 50 mm in x, y, and z axes, respectively.

## 3. Results

**Figure 3:**
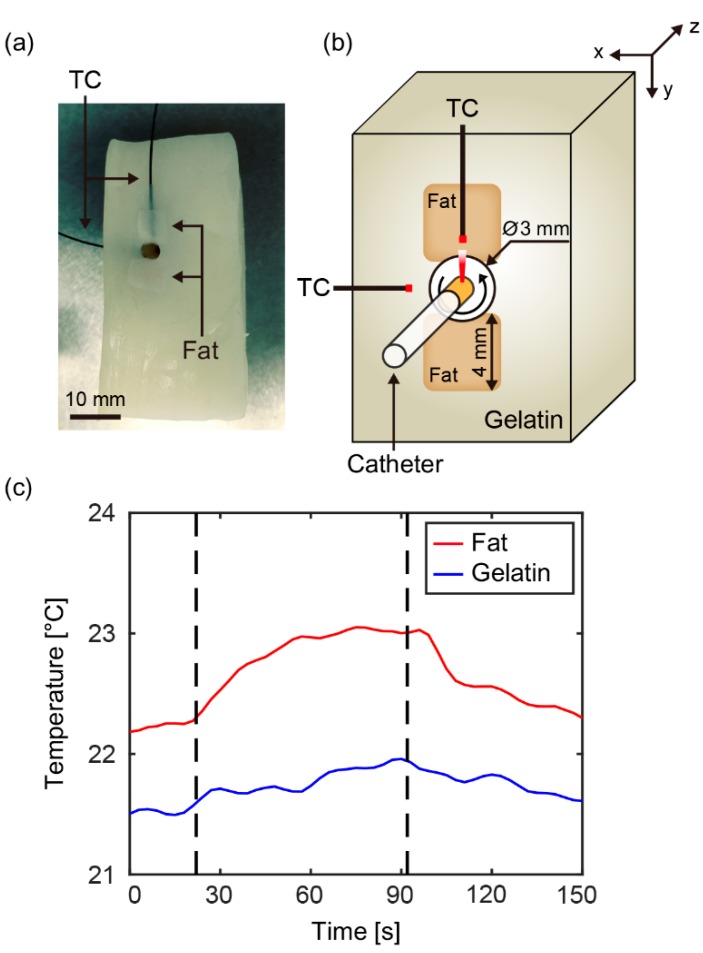
(**a**) A photograph of tissue-mimicking phantom. (**b**) A cross-sectional image of the catheter, phantom, and thermocouple at the image acquisition location. (**c**) A graph of temperature change of fat and gelatin, monitored by thermocouples. The laser was turned on at 22 s (a first dotted line), and off at 92 s (a second dotted line). TC, thermocouple.

To demonstrate the feasibility of intravascular pTSI system, we performed pTSI experiments using the prefabricated phantom. Figure 3a shows a photograph of the phantom. The porcine fat occupied the top and bottom of the phantom hole, and gelatin occupied the other side of the hole. We placed the phantom in the water-tank, inserted the catheter into the phantom hole, and put the thermocouples into the fat and gelatin at the image acquisition location as shown in Figure 3b. Figure 3c is a graph showing temperature change of the fat and gelatin monitored by the thermocouples in the experiment. We turned on the laser at 22 s (a first dotted line) and turned off at 92 s (a second dotted line). The laser heated the fat and gelatin inside of phantom holes with an output of 0.79 W/cm^2^. After heating, the gelatin temperature (indicated by the blue line) increased by 0.4 °C and the fat temperature (indicated by the red line) increased by 0.9 °C.

**Figure 4:**
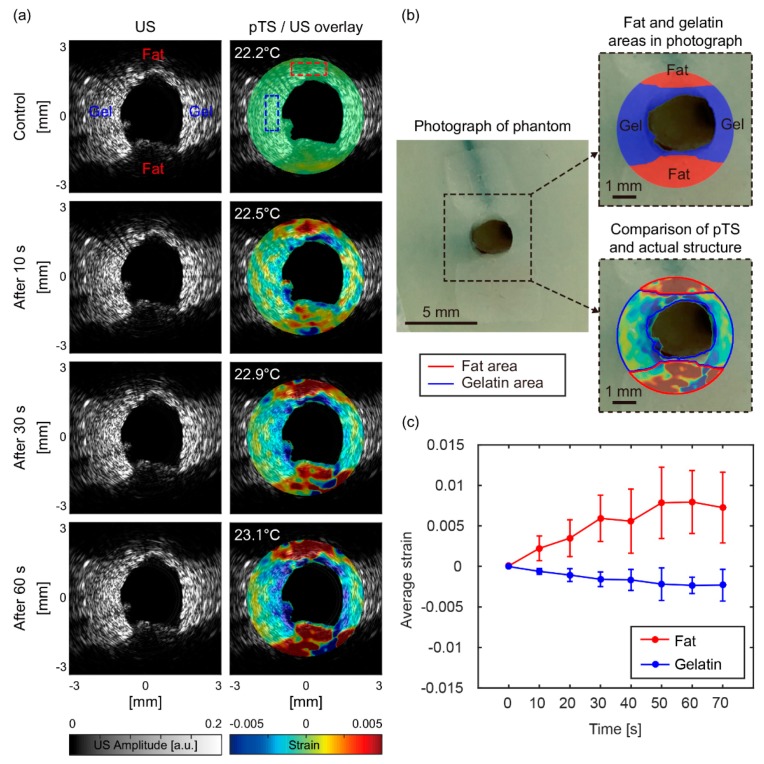
(**a**) Ultrasound images (1st column) and photothermal strain/ultrasound overlay images (2nd column) of the phantom at the time increased. (**b**) A photograph of phantom, an actual structural image of fat and gelatin areas, and a comparison image of the pTS image (at 60 s) and the actual structure image. (**c**) A graph of average strain change in fat (red dotted box in Figure 4a) and gelatin (blue dotted box in Figure 4a) over time. Gel, gelatin; US, ultrasound; pTS, photothermal strain.

Figure 4a shows the US images (1st column) and pTS/US overlay images (2nd column) when zero (control), 10, 30, and 60 s had passed since the laser was turned on. During the temperature change of about 1 °C, the changes in the US images due to speckle shifts were difficult to see visually. On the other hand, the strain changes in the overlay images were definitely changing, so we could perceive the changes sufficiently. First, the positive strain (indicated by the red area) appeared at the location of the fat and continued to increase as the temperature rose. In addition, the moderate negative strain (indicated by the blue area) appeared at the location of gelatin during the rise of temperature, which enabled us to distinguish between gelatin and fat. Figure 4b is a photograph of the phantom showing the structure of the fat and gelatin near the hole where the pTS catheter was inserted. Within the region where the pTS image was obtained, we were able to roughly distinguish the fat and gelatin areas in the photograph. Next, we compared the pTS image (at 60 s) with the photograph to see how well the strain matched the actual structure. First, we could see that the shape of the pTS area was very similar to the actual shape. It was also confirmed that the positive and negative strain of the pTS image closely matched to the fat and gelatin areas of the photograph. This result suggested that the location of fat could be found by analyzing the pTS image. Figure 4c shows the average strain in the area of fat (red dotted box Figure 4a) and gelatin (blue dotted box in Figure 4a) over time. It was confirmed that the change of average strain showed a reasonable tendency because the absolute value of λ in fat was approximately 1.5 times larger than the absolute value of λ in water-bearing tissue [36]. The result shows that the 1210-nm CW laser delivered through the optical fiber can raise the temperature of lipid and water to generate the strain, demonstrating the possibility of intravascular pTSI for lipid detection.

## 4. Discussion

The proposed intravascular pTSI has several advantages over other methods. First, pTSI can distinguish lipids based on optical properties. Unlike IVUS or IV-OCT, which lack the ability to detect lipids and are heavily dependent on user skills and experience, the ability to visualize lipids is a major benefit of pTSI. In addition, because pTSI can measure the image depth, it can identify lipids more effectively than other methods that provide information that can only indicate the presence of lipids. In terms of safety, pTSI is less burdensome to patients because it does not require the use of any chemicals, unlike many imaging techniques that use chemicals [40]. Because the laser can heat locally the target site, pTSI can also minimize unnecessary heating of the nontarget. Finally, the pTSI system uses relatively inexpensive and easily available CW lasers compared to pulsed lasers commonly used in many optical imaging methods. The pulsed lasers with wavelength for lipid detection are particularly difficult to obtain, so pTSI systems have the advantage of being relatively easy to configure using CW lasers.

However, some issues must be overcome before pTSI can be applied to clinical trials. The first problem is an image error that occurs because the starting positions do not match during rotation. Because the catheter rotation was inevitable in the current system, we reduced the rotation rate to minimize errors caused by this problem. We also applied the 2D cross-correlation-based position-correction algorithm in postprocessing to ensure that the starting positions of US images match as closely as possible. Local motion errors of the strain images are also a problem. We minimized motion errors due to vibration by limiting the surrounding environment as much as possible. In clinical trials, however, heartbeats can cause more motion errors than vibrations in the surrounding environment. We expect to be able to solve this problem using an array-type transducer and motion compensation algorithms. The array-type transducer can collect multiple signals at once, so images can be acquired very quickly and the motion effect can be minimized [41]. The array-type transducer could also reduce the angle mismatch problem because it does not require rotation [42]. In addition, the motion compensation algorithms such as electrocardiogram (ECG) gating [43] or motion artifact reduction [44] could be used to reduce errors generated by motion. Another problem with pTSI is the long heating time required to raise the temperature. If the laser power is close to the power limit that is acceptable when acquiring images within a few seconds, 2–3 s is sufficient to raise the tissue temperature by 1 °C. However, the current system takes a long time to heat the target because the laser is transmitted in only one direction during rotation. As a result, as the heating time became longer and the influence of heat conduction increased, the pTS images became relatively blurred. To overcome this problem, the optical path can be optimized to deliver the laser more efficiently using certain optical components. For example, using a micro cone-shaped mirror that reflects light in a 360° direction, the laser can heat a large area, as well as continuously heat the target area regardless of rotation [41,45]. This approach could reduce thermal conduction due to the temperature difference around the target while delivering more energy in a short time with the laser of the higher power.

The last issue to be solved is that the relationship between the degree of atherosclerosis and amount of strains produced has not been well studied and there is no gold standard for strain produced in the plaque lipid. To demonstrate this, follow-up studies should be conducted quantitatively. Based on the above discussion, the next objective will be the development of a clinical intravascular pTSI catheter using the array-type transducer and an improved optical pathway.

## 5. Conclusions

We developed a novel intravascular pTSI system using the 1210-nm laser to overcome the limitations of conventional intravascular imaging methods that struggle to image the plaque lipid. The catheter with the optical pathway was developed to deliver the laser beam and US at the same time. To establish the feasibility of the pTSI system, we performed the experiment using the tissue-mimicking phantom made of porcine fat and gelatin. By observing the strain that occurred while the laser heated the phantom in the hole, we successfully distinguished the fat from the gelatin. This result suggests that the intravascular pTSI system can be used to detect lipids that need to evaluate the risk of plaque rupture. If the above-discussed problems are solved and clinically available conditions are established, intravascular pTSI could be used in combination with conventional methods to potentially improve the survival probability of CVD patients. We expect pTSI to be a new approach to advancing intravascular imaging technology.
